# Relationship Between Patient Engagement and Depressive Symptoms Among People Living With HIV in a Mobile Health Intervention: Secondary Analysis of a Randomized Controlled Trial

**DOI:** 10.2196/20847

**Published:** 2020-10-29

**Authors:** Yu Zeng, Yan Guo, Linghua Li, Y Alicia Hong, Yiran Li, Mengting Zhu, Chengbo Zeng, Hanxi Zhang, Weiping Cai, Cong Liu, Shaomin Wu, Peilian Chi, Aliza Monroe-Wise, Yuantao Hao, Rainbow Tin Hung Ho

**Affiliations:** 1 Department of Medical Statistics School of Public Health Sun Yat-sen University Guangzhou China; 2 Sun Yat-sen Center for Global Health Guangzhou China; 3 Department of Infectious Diseases Guangzhou Number Eight People’s Hospital Guangzhou China; 4 Department of Health Administration and Policy College of Health and Human Services George Mason University Fairfax, VA United States; 5 South Carolina SmartState Center of Healthcare Quality Arnold School of Public Health University of South Carolina Columbia, SC United States; 6 Department of Health Promotion, Education, and Behavior Arnold School of Public Health University of South Carolina Columbia, SC United States; 7 National Center of AIDS/STD Control and Prevention Chinese Center for Disease Control and Prevention Beijing China; 8 Department of Psychology Faculty of Social Sciences University of Macau Macau China; 9 Department of Global Health University of Washington Seattle, WA United States; 10 Department of Social Work & Social Administration The University of Hong Kong Hong Kong China; 11 Centre on Behavioral Health The University of Hong Kong Hong Kong China

**Keywords:** mHealth, patient engagement, latent growth curve model, depressive symptoms, HIV

## Abstract

**Background:**

Associations between higher levels of patient engagement and better health outcomes have been found in face-to-face interventions; studies on such associations with mobile health (mHealth) interventions have been limited and the results are inconclusive.

**Objective:**

The objective of this study is to investigate the relationship between patient engagement in an mHealth intervention and depressive symptoms using repeated measures of both patient engagement and patient outcomes at 4 time points.

**Methods:**

Data were drawn from a randomized controlled trial (RCT) of an mHealth intervention aimed at reducing depressive symptoms among people living with HIV and elevated depressive symptoms. We examined the association between patient engagement and depressive symptoms in the intervention group (n=150) where participants received an adapted cognitive-behavioral stress management (CBSM) course and physical activity promotion on their WeChat social media app. Depressive symptoms were repeatedly measured using the Patient Health Questionnaire (PHQ-9) at baseline and 1 month, 2 months, and 3 months. Patient engagement was correspondingly measured by the completion rate, frequency of items completed, and time spent on the program at 1 month, 2 months, and 3 months. Latent growth curve models (LGCMs) were used to explore the relationship between patient engagement and depressive symptoms at multiple time points in the intervention.

**Results:**

The mean PHQ-9 scores were 10.2 (SD 4.5), 7.7 (SD 4.8), 6.5 (SD 4.7), and 6.7 (SD 4.1) at baseline, 1 month, 2 months, and 3 months, respectively. The mean completion rates were 50.6% (SD 31.8%), 51.5% (SD 32.2%), and 50.8% (SD 33.7%) at 1, 2, and 3 months, respectively; the average frequencies of items completed were 18.0 (SD 14.6), 32.6 (SD 24.8), and 47.5 (SD 37.2) at 1, 2, and 3 months, respectively, and the mean times spent on the program were 32.7 (SD 66.7), 65.4 (SD 120.8), and 96.4 (SD 180.4) minutes at 1, 2, and 3 months, respectively. LGCMs showed good model fit and indicated that a higher completion rate (β at 3 months=–2.184, *P*=.048) and a greater frequency of items completed (β at 3 months=–0.018, *P*=.04) were associated with fewer depressive symptoms at 3 months. Although not significant, similar trends were found in the abovementioned relationships at 1 and 2 months. There was no significant relationship between time spent on the program and depressive symptoms.

**Conclusions:**

This study revealed a positive association between patient engagement and health outcomes at 3 months of an mHealth intervention using LGCMs and repeated measures data. The results underscore the importance of improving patient engagement in mHealth interventions to improve patient-centered health outcomes.

**Trial Registration:**

Chinese Clinical Trial Registry ChiCTR-IPR-17012606; https://tinyurl.com/yxb64mef

**International Registered Report Identifier (IRRID):**

RR2-10.1186/s12889-018-5693-1

## Introduction

Mobile health (mHealth) has increasingly become a promising tool to deliver treatments for a range of psychosocial disorders [1-4]. Studies have shown that mHealth interventions are effective in reducing depressive symptoms among people living with HIV and elevated depressive symptoms [5-8]. In comparison with traditional face-to-face interventions, mHealth interventions have the potential to implement personalized and cost-effective interventions, provide real-time feedback, and overcome geographical and logistical barriers [9].

Despite the growing success of mHealth interventions, the relationship between patient engagement and intervention outcomes is still unclear. Patient engagement is defined as the extent to which participants actively interact with intervention content [10]. In face-to-face randomized controlled trials (RCTs), patient engagement has been found to be an essential predictor of intervention outcomes, with higher levels of patient engagement predicting better health outcomes of participants [11]. However, such a relationship has not yet been confirmed in mHealth interventions [12]. Findings regarding patient engagement and intervention outcomes in mHealth interventions are mixed. Some studies found a relationship similar to that observed in face-to-face interventions [13-18], while others found no significant relationship [19-21].

Understanding the relationship between patient engagement and health outcomes in mHealth interventions is critical for developing and implementing effective mHealth programs [12]. In most mHealth studies, patient engagement was only assessed at a single point in time and changes in patient outcomes were typically evaluated between pre- and postintervention [21]. There is a lack of examination of dose-response relationships between patient engagement and treatment outcomes during the entire course of the interventions. It is unclear whether such a dose-response relationship exists in mHealth interventions, and if yes, whether such a relationship occurs early in the intervention as demonstrated in some face-to-face interventions [22] or appears later in the intervention. Existing mHealth studies have varying intervention durations, which may lead to differential results of the dose-response relationship. For example, for the same purpose of reducing depressive symptoms among the general population, one mHealth intervention lasted 4 weeks whereas another was 24 weeks in duration [19,23]. With different lengths of treatment, it might be difficult to compare the dose-response relationship in these mHealth interventions, especially when the relationship was only evaluated pre- and postintervention. More studies are needed to explore the potentially changing relationship between patient engagement and health outcomes during the course of mHealth interventions. Longitudinal analyses with repeated measures of both patient engagement and treatment outcomes are likely to improve our understanding of the dose-response relationship throughout the course of an intervention.

To fill the gaps in the existing literature, this study aimed to explore the relationship between patient engagement and depressive symptoms during the 3-month course of an RCT of an mHealth program for people living with HIV and elevated depressive symptoms, the Run4Love intervention [24]. We used 3 measures of patient engagement to capture multiple aspects of this construct in the study, as suggested by a recent systematic review on adherence reporting in online RCTs [12]. Using 4 time points of outcome measures and the corresponding patient engagement measures, we examined the levels of and changes in patient engagement and depressive symptoms at 1, 2, and 3 months and the relationship between patient engagement and depressive symptoms at 1, 2, and 3 months.

## Methods

### Study Setting

This study is a secondary analysis of data from the intervention group of a 3-month RCT (Chinese Clinical Trial Registry [ChiCTR-IPR-17012606]) that was conducted to examine the efficacy of a WeChat-based intervention on reducing depressive symptoms among people living with HIV and elevated depressive symptoms [8]. WeChat, similar to WhatsApp, is a widely used social media platform for instant communication in China with 1.1 billion active users [25]. Participants were recruited from the outpatient department of a large hospital designated for HIV treatment in 2017 in Guangzhou, the third largest city in China, with a population of more than 13 million [26]. A total of 300 people living with HIV who had elevated depressive symptoms (Center for Epidemiological Studies Depression Scale [CES-D] score ≥16) and used WeChat were randomized into the intervention or wait-list control group in a 1:1 ratio. The institutional review board of Sun Yat-sen University approved the intervention protocol, and the detailed recruitment and randomization procedure were described elsewhere [24].

### Intervention

In addition to routine care, the Run4Love intervention consisted of two major components, an adapted cognitive-behavioral stress management (CBSM) course and physical activities promotion. The adapted CBSM course included content covering coping skills, muscle relaxation, and meditation tutorials. The physical activities promotion consisted of information on the benefits of regular exercise, exercise guidance, and healthy dietary suggestions. During the 3-month intervention, multimedia items were delivered to participants to support the adapted CBSM course and physical activities promotion via WeChat in the form of short articles, audio recordings, and posters almost once a day (5 to 6 items per week). Out of 65 items in total, 29 were short articles with an average of 1300 words, requiring about 5 minutes to read; 24 were posters with motivational messages, requiring about 30 seconds to read; and 12 were audio recordings in Chinese requiring 5 to 10 minutes to listen to. The WeChat-based Run4Love platform with extended functions was used to automatically deliver intervention items and collect real-time data on patient engagement (eg, time spent on an article, audio recording, or poster). The intervention design has been detailed elsewhere [24].

During the intervention, we used several measures to promote engagement: (1) automatically sending feedback on each participant’s compliance via WeChat on a weekly basis (eg, number of items completed and not completed); (2) giving verbal encouragement and financial incentives (up to US $2) via WeChat on a weekly basis based on the level of compliance; and (3) conducting motivational interviews by phone to sustain participation and help overcome barriers of compliance at 1 week, 1 month, and 2 months after baseline.

### Measurement

#### Depressive Symptoms

Depressive symptoms were assessed by the Chinese version of the Patient Health Questionnaire (PHQ-9) at baseline, 1 month, 2 months, and 3 months [27]. Compared with the 20-item measurement of CES-D, PHQ-9 is shorter but has demonstrated both good reliability and validity in the Chinese populations [28,29]. The scale consists of 9 items such as “Little interest or pleasure in doing things” and “Feeling down, depressed, or hopeless” to measure the depressive status of the participants over the previous 2 weeks. Each item was rated on a 4-point Likert scale ranging from 0 (not at all) to 3 (nearly every day), providing a 0 to 27 total severity score, with higher scores indicating increased depressive symptoms [30]. A good reliability of PHQ-9 was shown in the study, and the Cronbach alphas were .814, .861, .872, and .852 at baseline, 1 month, 2 months, and 3 months, respectively.

#### Patient Engagement

Three cumulative measures, including completion rate, frequency of items completed, and time spent on the program, were used to assess patient engagement in this study. Completion rate was the ratio of the number of items a patient completed to the number of items assigned, where the items that had been clicked were considered completed. Frequency of items completed was assessed not only by the number of items read or listened to by the participants, but also by the number of times the items were repeatedly read or listened to. Compared with the completion rate, frequency of items completed captured the repeat aspect of patient engagement. For example, if the same item was read or listened to twice by a participant, frequency was counted as 2 and the frequency of items completed for the participant was the accumulative frequency for all the items. The time spent on the program was measured by the overall time a participant spent on the Run4Love program, including reading or listening to the delivered items during the course of the intervention. We repeatedly assessed these 3 measures of patient engagement in conjunction with measurements of depressive symptoms that were evaluated at 1, 2, and 3 months after baseline, as has been recommended in previous studies [31]. Specifically, all 3 measures at 1, 2, and 3 months were used to assess the average patient engagement from baseline until 1, 2, and 3 months after baseline.

#### Baseline Characteristics

Sociodemographic characteristics and HIV-related variables were collected at baseline including age, gender, sexual orientation, education, marital status, employment status, income, and duration of HIV infection (years). Income was measured by asking the participants whether they had adequate income to cover their daily expenses (adequate or inadequate).

### Statistical Analysis

Descriptive statistics on depressive symptoms, 3 measures of patient engagement, and baseline characteristics were provided. Mean and standard deviation were used to describe continuous variables with normal distribution, median and interquartile range (IQR) for continuous variables that did not follow a normal distribution, and frequency and percentage for categorical variables. Latent growth curve models (LGCMs) were constructed to examine the time-varying associations between patient engagement and depressive symptoms using repeated measure data at 4 time points [32]. Maximum likelihood estimation with robust standard errors (MLR) was used to handle missing data and obtain parameter estimates [32]. Descriptive analyses were conducted in SPSS Statistics version 20.0 (IBM Corporation), and LGCMs were conducted in Mplus version 7 (Muthen & Muthen).

The analyses of LGCMs were conducted in two steps [32]. First, we developed an unconditional LGCM to estimate the growth trajectory of depressive symptoms across time. The initial level of depressive symptoms was represented as the intercept, and the average rate of change was represented as the slope in the model. For the intercept factor, all loadings were fixed to 1. For the slope factor, the first and second loadings were fixed to 0 and 1, and the remaining loadings were freely estimated from the data to test whether there was a linear or nonlinear growth trajectory (eg, linear growth pattern: 0, 1, 2, 3; nonlinear growth pattern: the third and fourth loadings significantly differ from 2 and 3, respectively).

Second, we developed 3 conditional LGCMs to examine the effects of patient engagement on depressive symptoms based on the 3 measures of patient engagement. The conditional model was extended from the unconditional model to incorporate one measure of patient engagement and related baseline characteristics as covariates. Since no baseline characteristics were found to significantly associate with the 3 measures of patient engagement or the outcome of depressive symptoms in this study, we included variables such as education, income, and duration of HIV infection that were associated with depressive symptoms among people living with HIV in the literature as control variables in the conditional models [33-35]. Associations between patient engagement and depressive symptoms were treated as time-varying effects, which were specified by regressing depressive symptoms (eg, PHQ-9 at 1 month) on the corresponding patient engagement variable (eg, completion rate at 1 month). Patient engagement, therefore, could be considered as a time-specific predictor of depressive symptoms after controlling for the influences of the underlying growth trajectory of depressive symptoms and baseline characteristics [32].

Model fit of LGCM was evaluated using chi-square tests and indices including the standardized root mean of residual (SRMR), confirmatory fit index (CFI), Tucker-Lewis index (TLI), and root mean-squared error of approximation (RMSEA). The criteria used to determine adequate model fit included: CFI≥0.95, TLI≥0.95, SRMR≤0.08, and RMSEA≤0.08 [36-38].

## Results

### Baseline Characteristics

As shown in Table 1, the mean age of participants was 28.0 (SD 5.8) years and the average duration of HIV infection was 2.4 (SD 2.3) years. The participants were predominately male (142/150, 94.7%), nonheterosexual (130/150, 86.7%), well-educated (98/150, 65.3%, with at least some college education), single (132/150, 88.0%), employed (123/150, 82.0%), and had adequate income to cover daily expenses (129/150, 86.0%).

**Table 1:** Baseline characteristics of people living with HIV and elevated depressive symptoms in the intervention group (n=150).

Variables		Value
Age in years, mean (SD)		28.0 (5.8)
**Gender, n (%)**		
	Male	142 (94.7)
	Female	8 (5.3)
**Sexual orientation, n (%)**		
	Heterosexual	20 (13.3)
	Homosexual, bisexual, uncertain	130 (86.7)
**Education, n (%)**		
	Below or at high school level	52 (34.7)
	Above high school level	98 (65.3)
**Marital status, n (%)**		
	Single, divorced, widowed	132 (88.0)
	Married	18 (12.0)
**Employment status, n (%)**		
	Unemployed	27 (18.0)
	Employed	123 (82.0)
**Income, n (%)**		
	Adequate to cover daily expenses	129 (86.0)
	Inadequate to cover daily expenses	21 (14.0)
Duration of HIV infection in years, mean (SD)		2.4 (2.3)

### Change in Depressive Symptoms and Patient Engagement Over Time

Of the participants, 87.3% (131/150), 76.0% (114/150), and 92.0% (138/150) completed the online questionnaire for depressive symptoms at 1, 2, and 3 months, respectively, after baseline. Compared with the depressive symptom score at baseline (PHQ-9: mean 10.2 [SD 4.5]), all follow-up assessments reported a decrease in depressive symptoms with mean values of 7.7 (SD 4.8), 6.5 (SD 4.7), and 6.7 (SD 4.1) at 1, 2, and 3 months, respectively (Table 2).

Nearly all participants (149/150) read or listened to at least one item during the 3-month intervention. A moderate level of patient engagement was found during the intervention process. The completion rate remained around 50.0% (33/65) during the course of the intervention, with a mean of 50.6% (SD 31.8%), 51.5% (SD 32.2%), and 50.8% (SD 33.7%) at 1, 2, and 3 months, respectively. The mean frequency of items completed was 18.0 (SD 14.6), 32.6 (SD 24.8), and 47.5 (SD 37.2) at 1, 2, and 3 months, respectively. The mean time spent on the program was 32.7 (SD 66.7), 65.4 (SD 120.8), and 96.4 (SD 180.4) minutes at 1, 2, and 3 months, respectively. When comparing the mean and median scores in Table 2, we found that time spent on the program was positively skewed, with means much larger than the medians at 1, 2, and 3 months.

**Table 2:** Change in depressive symptoms and patient engagement across the 3-month intervention^a^.

Variables									
	Baseline		1 month		2 months		3 months		
	Mean (SD)	Median (IQR)	Mean (SD)	Median (IQR)	Mean (SD)	Median (IQR)		Mean (SD)	Median (IQR)
PHQ-9^b^	10.2 (4.5)	10.0 (7.0-13.0)	7.7 (4.8)	8.0 (4.0-11.0)	6.5 (4.7)	6.0 (3.0-9.0)		6.7 (4.1)	7.0 (3.0-9.0)
Completion rate (%)	—^c^	—	50.6 (31.8)	54.2 (16.7-81.8)	51.5 (32.2)	53.0 (20.6-81.7)		50.8 (33.7)	47.6 (16.9-85.3)
Frequency of items completed	—	—	18.0 (14.6)	17.0 (5.8-26.0)	32.6 (24.8)	29.5 (10.0-51.3)		47.5 (37.2)	41.0 (11.0-78.3)
Time spent on program (min)	—	—	32.7 (66.7)	9.7 (2.5-42.1)	65.4 (120.8)	22.0 (5.3-80.3)		96.4 (180.4)	31.3 (6.7-117.2)

^a^For PHQ-9, the sample sizes were 150, 131, 114, and 138 at baseline, 1 month, 2 months, and 3 months, respectively. For patient engagement, the sample size remained 150 across the 3-month intervention.

^b^PHQ-9: Patient Health Questionnaire.

^c^Not available.

### Unconditional Latent Growth Curve Model

**Figure 1:**
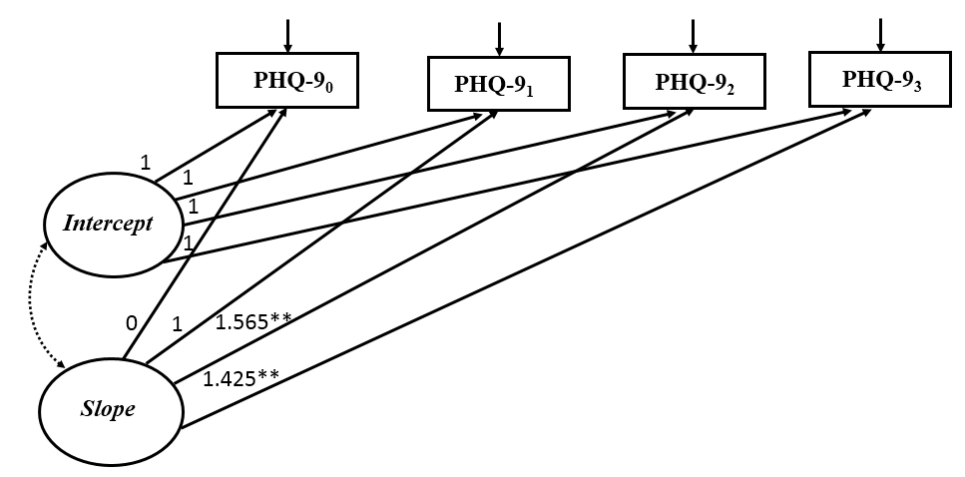
Unconditional latent growth curve model for depressive symptoms (n=150). Model fit statistic: *χ*^2^=7.0, *P*=.07, degrees of freedom=3; CFI=0.975, TLI=0.949, SRMR=0.039, RMSEA=0.094. Observed variables were indicated by boxes and latent variables were indicated by ovals. Unidirectional arrows indicated the effect of one variable on another and bidirectional arrows indicated correlations. The nonsignificant path was indicated by dotted line. 0: baseline; 1: 1 month after baseline; 2: 2 months after baseline; 3: 3 months after baseline; *P<.05, **P<.001.

Results of the unconditional model indicated that depressive symptoms decreased during the 3-month intervention, and the largest reduction occurred at 1 month after baseline. This model had good model fit (Figure 1). The mean intercept was 10.157 (*P*<.001), indicating a mean PHQ-9 score of 10.157 at baseline. The mean slope was –2.336 (*P*<.001), and the factor loadings on the slope were 0, 1, 1.565, and 1.425 at baseline, 1 month, 2 months, and 3 months, respectively. These results indicated a nonlinear pattern of the reduction of depressive symptoms: a rapid decrease of PHQ-9 score (2.336 points) occurred at the first month, and then the magnitude of reduction flattened out. Variances of the intercept and slope were 15.069 (*P*=.003) and 5.564 (*P*=.12), indicating significant individual differences in depressive symptoms at baseline, but no individual differences in the rates of change over time. Covariance between the intercept and slope was –4.381 (*P*=.25), suggesting that the rate of change in depressive symptoms was independent of its initial levels (Table 3).

**Table 3:** Parameter estimates for unconditional latent growth curve model for depressive symptoms (n=150).

Growth parameters	Estimate	SE	*P* value
Slope_0_→PHQ-9_0_^a^	0.000	0.000	—^b^
Slope_1_→PHQ-9_1_	1.000	0.000	—
Slope_2_→PHQ-9_2_	1.565	0.196	<.001
Slope_3_→PHQ-9_3_	1.425	0.225	<.001
Mean intercept	10.157	0.370	<.001
Mean slope	–2.336	0.411	<.001
Variance of intercept	15.069	5.100	.003
Variance of slope	5.564	3.614	.12
Covariance of intercept and slope	–4.381	3.799	.25

^a^PHQ-9: Patient Health Questionnaire.

^b^Not available.

### Conditional Latent Growth Curve Models

**Figure 2:**
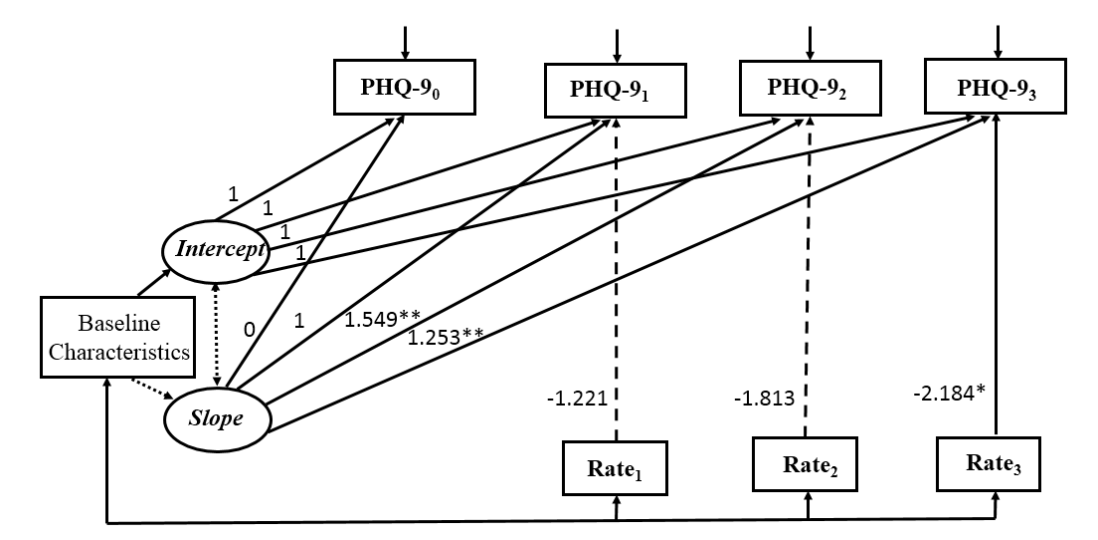
Conditional latent growth curve model for depressive symptoms with completion rate as the measure of patient engagement (n=150). Model fit statistic: *χ*^2^=13.0, *P*=.79, degrees of freedom=18; CFI=1.000; TLI=1.000; RMSEA<0.001; SRMR=0.030. Baseline characteristics included education, income, and duration of HIV infection. Observed variables were indicated by boxes and latent variables were indicated by ovals. Unidirectional arrows indicated the effect of one variable on another and bidirectional arrows indicated correlations. Nonsignificant paths were indicated by dotted lines. Rate: completion rate; 0: baseline; 1: 1 month after baseline; 2: 2 months after baseline; 3: 3 months after baseline; *P<.05, **P<.001.

**Figure 3:**
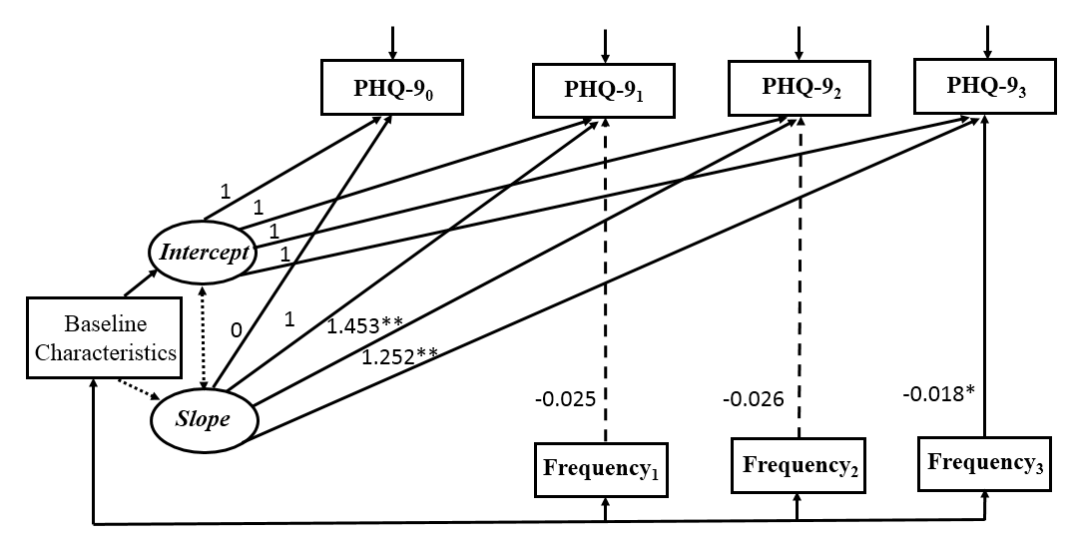
Conditional latent growth curve model for depressive symptoms with frequency of items completed as the measure of patient engagement (n=150). Model fit statistic: *χ*^2^=14.0, *P*=.73, degrees of freedom=18; CFI=1.000; TLI=1.000; RMSEA<0.001; SRMR=0.028. Baseline characteristics included education, income, and duration of HIV infection. Observed variables were indicated by boxes and latent variables were indicated by ovals. Unidirectional arrows indicated the effect of one variable on another and bidirectional arrows indicated correlations. Nonsignificant paths were indicated by dotted lines. Frequency: frequency of items completed; 0: baseline; 1: 1 month after baseline; 2: 2 months after baseline; 3: 3 months after baseline; *P<.05, **P<.001.

**Figure 4:**
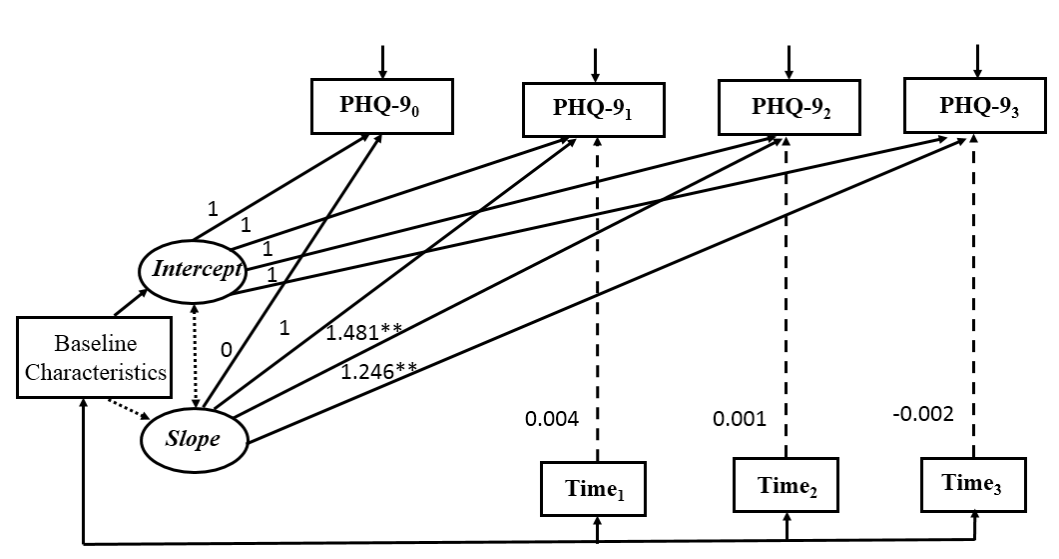
Conditional latent growth curve model for depressive symptoms with the time spent on the program as the measure of patient engagement (n=150). Model fit statistic: *χ*^2^=19.6, *P*=.36, degrees of freedom=18; CFI=0.993; TLI=0.988; RMSEA=0.024; SRMR=0.025. Baseline characteristics included education, income, and duration of HIV infection. Observed variables were indicated by boxes and latent variables were indicated by ovals. Unidirectional arrows indicated the effect of one variable on another and bidirectional arrows indicated correlations. Nonsignificant paths were indicated by dotted lines. Time: time spent on the program; 0: baseline; 1: 1 month after baseline; 2: 2 months after baseline; 3: 3 months after baseline; *P<.05, **P<.001.

Three conditional LGCMs were constructed, each using one measure of patient engagement as a time-varying variable. Results of the conditional models indicated that higher completion rate and greater frequency of items completed were significantly associated with lower depressive symptoms at 3 months, but there was no significant relationship between time spent on the program and depressive symptoms over the course of the intervention. All conditional models reported good model fit (Figures 2-4).

Based on the literature, baseline characteristics of education, income to cover daily expenses, and duration of HIV infection were included as control variables. As shown in Table 4, education and duration of HIV infection did not have significant effect either on the initial levels (intercept) or the rate of change (slope) of depressive symptoms in all conditional models. Income had a significant effect on the intercept in all conditional models, suggesting that participants with adequate income to cover daily expenses had a lower level of depressive symptoms at baseline. The time-varying variable, patient engagement, had a significant effect on depressive symptoms at 3 months after baseline (completion rate: β3=–2.184, *P*=.048; frequency of items completed: β3=–0.018, *P*=.04), whereas the effect of patient engagement, regardless of the measurement, was not significant on depressive symptoms at 1 or 2 months. Specifically, participants who had higher completion rates or greater frequency of items completed were more likely to report fewer depressive symptoms at 3 months after controlling for the underlying growth trajectory of depressive symptoms and baseline characteristics. By contrast, the time spent on the program was not significantly associated with depressive symptoms throughout the intervention process (β1=0.004, *P*=.29; β2=–0.001, *P*=.74; β3=–0.002, *P*=.16), suggesting that the amount of time patients spent on the intervention program was not related to the changes in depressive symptoms.

**Table 4:** Parameter estimates for the three conditional latent growth curve models (n=150).

Covariate parameters		Completion rate, estimate (SE)	Frequency of items completed, estimate (SE)	Time spent on program, estimate (SE)
**Baseline characteristics**				
	Education→intercept	–0.893 (0.858)	–0.914 (0.849)	–0.909 (0.843)
	Income→intercept	–2.329^b^ (1.179)	–2.306^b^ (1.175)	–2.309^b^ (1.169)
	Duration of HIV infection→intercept	0.064 (0.165)	0.058 (0.157)	0.061 (0.156)
	Education→slope	–0.257 (0.650)	–0.274 (0.632)	–0.239 (0.628)
	Income→slope	1.382 (0.860)	1.298 (0.899)	1.399 (0.898)
	Duration of HIV infection→slope	0.103 (0.155)	0.116 (0.147)	0.092 (0.139)
**Measures of patient engagement**				
	Engagement_1_→PHQ-9_1_^a^	–1.221 (0.881)	–0.025 (0.019)	0.004 (0.004)
	Engagement_2_→PHQ-9_2_	–1.813 (1.158)	–0.026 (0.014)	0.001 (0.002)
	Engagement_3_→PHQ-9_3_	–2.184^b^ (0.945)	–0.018^b^ (0.009)	–0.002 (–0.082)

^a^PHQ-9: Patient Health Questionnaire.

^b^*P*<.05.

## Discussion

### Principal Findings

This study was among the first to explore dose-response relationship between patient engagement and depressive symptoms in a social media–based mHealth intervention among people living with HIV using longitudinal data. Three measures of patient engagement were used to assess different aspects of the construct. By investigating the time-varying relationship between patient engagement and health outcomes, our study provided a better understanding of the dose-response relationship in mHealth interventions and the time of occurrence of this association. Results of LGCMs revealed that two measures of patient engagement, completion rate and frequency of items completed, were significantly associated with reduced depressive symptoms, and these relationships occurred at 3 months of the intervention but not at 1 or 2 months. However, time spent on the program was not significantly related to depressive symptoms throughout the intervention.

First and foremost, this study provides new evidence on the dose-response relationship in mHealth interventions as the literature in this regard was limited and inconclusive [39]. Potential reasons for the ambivalent relationship in previous mHealth intervention studies could include inadequate research design or implementation, inadequate intervention durations for significant treatment effect, low levels of patient engagement, high dropout rate, and/or inappropriate or inadequate measurements of patient engagement or data analysis methods [12,20,39]. This study, however, shows that an evidence-based mHealth intervention with rigorous design and implementation could produce significant treatment effects, in particular, for participants with better engagement.

Our data suggests that the significant relationship of patient engagement and health outcomes did not occur early (1 or 2 months) in the intervention, but only at 3 months. Few studies have explored the dose-response relationship across the span of an intervention, especially in mHealth programs. To our knowledge, only one mHealth study intended to explore the patient engagement–outcome relationship using multiple measures of patient engagement [13]. However, the web-based iCBT intervention conducted in a community setting in Dublin, Ireland, in 2014 only measured pre- and postintervention depressive symptoms [13]. The methodology of repeated-measures analysis of variance used in the study could only reveal that patients with significant improvement in depressive symptoms had higher levels of patient engagement during the 8-week intervention [13]. Although the study further found that patients tended to engage in the online program more in the first half of the program (1 to 4 weeks) than in the latter half (5 to 8 weeks) [13], the design and findings of the study could not identify when the linkage between patient engagement and outcome occurred during the course of the intervention.

Compared with mHealth interventions, face-to-face studies have focused more on the relationship between patient engagement and health outcomes. One face-to-face intervention investigated the dose-response relationship in the first 5 weeks of the intervention using both weekly engagement measures and depressive symptoms outcomes among people with major depressive disorder [22]. However, the study used repeated measures regression analysis that could only conclude that there was a significant dose-response relationship during the 5-week intervention but not in which week(s) the relationship started to appear. Another face-to-face study conducted among patients with diabetes in Netherlands investigated the dose-response relationship between patient engagement and depressive symptoms during an 8-week intervention [40]. Patient engagement and depressive symptoms were repeatedly measured at 2, 4, and 8 weeks [40]. Although the study used multilevel analysis that was able to identify both the dose-response relationship and timing of the occurrence of any relationship, the study did not find a dose-response relationship in any of the time points [40]. One potential explanation for the nonsignificant dose-response relationship might be that individuals with chronic diseases and depressive symptoms need a longer time of exposure to an intervention to obtain the beneficial outcomes. Likewise, our study did not find a dose-response relationship at 1 or 2 months of the intervention but only at 3 months of the intervention among people living with HIV and depressive symptoms. Such a relationship among individuals with chronic diseases and psychological disorders needs to be further explored in future studies. Although the nonsignificant dose-response relationship at 1 or 2 months seemed to be contradictory to the finding that most reduction of depressive symptoms occurred at 1 month, reasons for the reduction of depressive symptoms could be many. Besides patient engagement, other factors such as social support might also play a role in the reduction of depressive symptoms. For example, several phone calls made by research staff to motivate participants to continue the intervention at 1 week and 1 and 2 months after baseline may provide social support needed by people living with HIV and elevated depressive symptoms.

Identifying the time point of effective dose-response relationship between patient engagement and treatment outcomes is critical for optimizing the design, pace, and duration of interventions. Unnecessarily lengthy interventions waste resources and lead to high dropout rates [41], whereas interventions with inadequate duration may not generate intended outcomes [42]. Repeatedly examining the relationship between patient engagement and health outcomes allows us to identify the optimal time for intervention. This study demonstrates such measurement in an mHealth intervention.

In addition to identifying the timing of occurrence of the dose-relationship in the intervention, this study also found the differential relationships between different measures of patient engagement and health outcomes. Compared with face-to-face interventions, mHealth interventions have more measurement tools to capture different aspects of patient engagement [12]. These different measurements may not have similar impacts on patient outcomes and may explain the conflicting results regarding the dose-response relationships in mHealth interventions [12].

In this study, participants with higher completion rates reported fewer depressive symptoms at 3 months. On average, participants completed 50.8% (33/65) of the program items (ie, short articles, audio recordings, and posters) at 3 months, which was similar to the completion rates reported in other online interventions for depressive symptoms [10,13]. A recent meta-analysis found a significant association between higher completion rates and reduced depressive symptoms in online programs [43]. However, some more recent internet-based RCTs did not find such a relationship [19,20]. One potential reason for the nonsignificant relationship was low levels of patient engagement in these mHealth interventions [10]. Therefore, adherence to the program needs to be underscored in mHealth interventions, especially considering that most of the existing mHealth interventions have relatively low completion rates [10,13].

Frequency of items completed was another patient engagement measure we used in the study, and it was also positively associated with patients’ outcomes. Such a measure of patient engagement has rarely been reported in the existing mHealth interventions. At 3 months of the intervention, participants had read or listened to the items 47.5 times on average. As the average number of items completed by participants was 33, each item was repeatedly read or listened to for approximately 1.5 times (47.5/33). This measure captured an important aspect of patient engagement as it reflected to what extent participants were willing to repeatedly engage with the intervention items. Based on this frequency data, we subsequently chose items that had been most read or listened to and re-sent these items to the patients in a booster session 3 months after the intervention [8]. In a separate study, we found a continued intervention effect with further reductions of patients’ depressive symptoms at 3 and 6 months after the intervention [8].

In contrast, the third patient engagement measure, time spent on the program, was found not to relate to the reduced depressive symptoms, which was consistent with previous studies [21,44]. Time spent on the program may not reflect the actual levels of patient engagement as it is likely to be influenced by a variety of factors such as reading speed, cognitive capacity, and familiarity with the program [39]. Participants might keep the program on without actively engaging. For example, patients might fall asleep when listening to a relaxation audio recording; yet time stayed on the page was still counted as time spent on the program. In this study, there was a noticeable difference (31.3 vs 96.7 minutes) between the median and mean time spent on the platform at 3 months of the intervention, indicating a positively skewed distribution of patients’ time spent online, suggesting few patients spent extra-long time on the platform. Future interventions should incorporate a time-out function to better capture the actual patient engagement.

### Limitations

Several limitations in this study should be noted. First, although the sample was drawn from a large hospital designated for HIV treatment in Guangzhou with more than 10,000 HIV seropositive patients on treatment, participants were mostly from an urban setting and the majority of them were nonheterosexual young men. Thus, the generalizability of the findings to other people living with HIV or other populations may be limited. Second, although depressive symptoms are the major outcome in this study, more types of outcome measures such as other psychological indicators (eg, anxiety) and/or biomarkers (eg, hair or salivary cortisol concentration) can be incorporated in future studies to provide further evidence on dose-response relationship between patient engagement and intervention outcomes. Third, while measures of patient engagement were collected objectively through the Run4Love platform, depressive symptoms were self-reported and might suffer from social desirability and recall bias. Fourth, there might be measurement biases in assessing patient engagement. For example, when clicked, a material was readily considered as completed. Nevertheless, this definition of completion was widely used in mHealth interventions, and the relevant patient engagement measures served as reliable and efficient metrics to assess dose-response relationship [12]. Additionally, time spent on the program might not capture the real patient engagement as it was not able to differentiate between active and inactive engagement and time on distraction or interruption could not be measured. Finally, we did not differentiate patient engagement in different formats of the intervention items (ie, short articles, audio recordings, and posters) nor did we examine the relationship between the content of the Run4Love intervention and patient engagement in this study, as it goes beyond the purposes of this study. We call for future research to further explore the relationships between patient engagement and the content of the intervention as well as the potential differences in patient engagement by different types of multimedia items in mHealth interventions.

### Implications

This study indicated that higher completion rate or greater frequency of accessing items in an mHealth intervention contributed to reduced depressive symptoms among people living with HIV and elevated depressive symptoms at 3 months in the intervention. Given the observed positive impact of patient engagement on intervention outcomes, adherence to mHealth interventions should be evaluated and strengthened as a critical and integral strategy to improve treatment effects. Approaches for promoting patient engagement include enhancing patients’ initial motivation in program participation, education on the importance of active participation, frequent and effective reminders, financial and verbal incentives, and engaging patients in goal setting (eg, physical activities) [45-47]. Future studies with repeated assessments of patient engagement and outcomes throughout the intervention process have the potential to analyze the time-varying dose-response relationship. Such studies would optimize the intervention duration and maximize intervention outcomes through improving the design and implementation of mHealth interventions.

### Conclusion

In conclusion, this study revealed a positive association between patient engagement and reduction in depressive symptoms at 3 months of an mHealth intervention using latent growth curve models and repeated measure data from 4 time points. The results underscore the importance of enhancing patient engagement in mHealth interventions to improve health outcomes of the participants.

## Abbreviations

CBSM: cognitive-behavioral stress management course
CES-D: Center for Epidemiological Studies Depression Scale
CFI: confirmatory fit index
IQR: interquartile range
LGCM: latent growth curve model
mHealth: mobile Health
MLR: maximum likelihood estimation with robust standard error
PHQ-9: Patient Health Questionnaire
RCT: randomized controlled trial
RMSEA: root mean-squared error of approximation
SRMR: standardized root mean of residual
TLI: Tucker-Lewis index
